# Supplementary material for: Promotion of the Efficient Electrocatalytic Production of H2O2 by N,O- Co-Doped Porous Carbon
Source: Nanomaterials (Basel). 2023 Mar 27;13(7):1188. doi: 10.3390/nano13071188 (PMC10096704; doi:10.3390/nano13071188)
Supplement: Supplementary file 1 [file nanomaterials-13-01188-s001.zip › nanomaterials-2265623-supplementary.pdf]

Promotion the Efficient electrocatalytic production of H_2O_2 by N,O-co-doped porous carbon

Sun Lina [a,b], Sun Liping [a*], Huo Lihua [a], Zhao Hui[a*]

a Key Laboratory of Functional Inorganic Material Chemistry, Ministry of Education, School of Chemistry and Materials Science, Heilongjiang University, Harbin 150080, P. R. China

b Key Laboratory of Molten Salts and Functional Materials of Heilongjiang Province, School of Science, Heihe University, Heihe 164300, P. R. China

1. Quantification of H_2O_2

Based on the reduction of Ce^{4+} (yellow) to Ce^{3+} (colorless) by H_2O_2 in acidic solution, the H_2O_2 (HO_2^-) produced in bulk electrolysis was measured by $Ce(SO_4)_2$ titration method. The produced H_2O_2 is Quantitatively analyzed based on the following equation

$$2\,Ce^{4+} + H_2O_2 \longrightarrow 2\,Ce^{3+} + 2\,H^+ + O_2\uparrow$$

Based on the fact that the intensity of Ce^{4+} adsorption peak at 318 nm is proportional to its concentration, a series of $Ce(SO_4)_2$ solutions with known concentration are prepared and the standard curve is plotted by Ce^{4+} concentration vs. the intensity of adsorption peak. Next, to quantify the produced H_2O_2, a certain volume of sample solution is mixed with 0.5 mM Ce^{4+} solution. After standing for 2 h, the mixture solution is measured by UV-vis spectrophotometry. The yield of H_2O_2 is then determined based on the reduced Ce^{4+} concentration.

* Corresponding author. Tel.: +86 45186608426; fax: +86 45186608426.

E-mail address: sunliping@hlju.edu.cn (L-P. Sun), zhaohui98@hlju.edu.cn (H. Zhao)

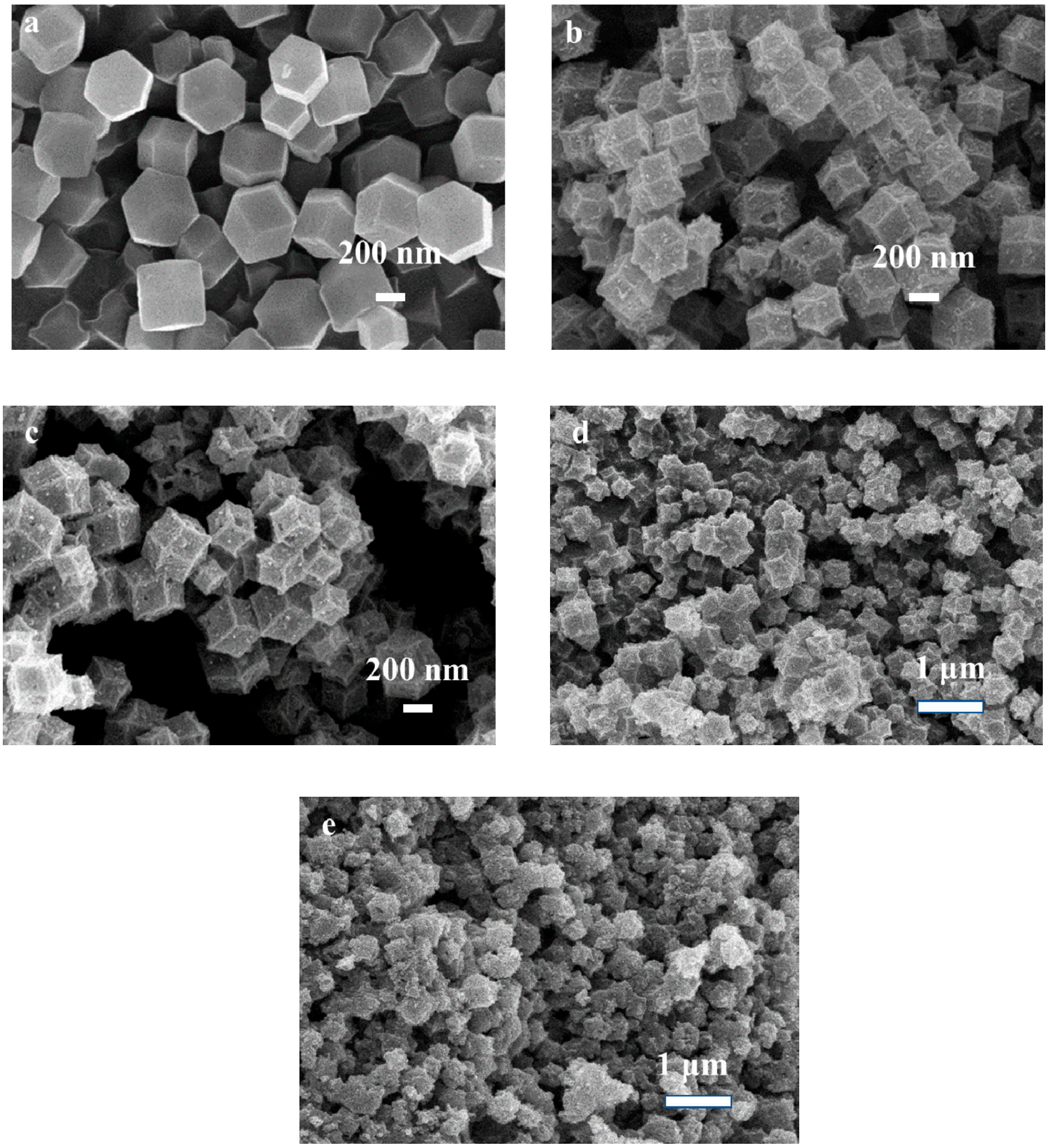

Figure S1 SEM image of the catalyst. (a) ZIF-67. (b) NPC-900. (c) O-NPC-80. (d) O-NPC-100. (e) O-NPC-120.

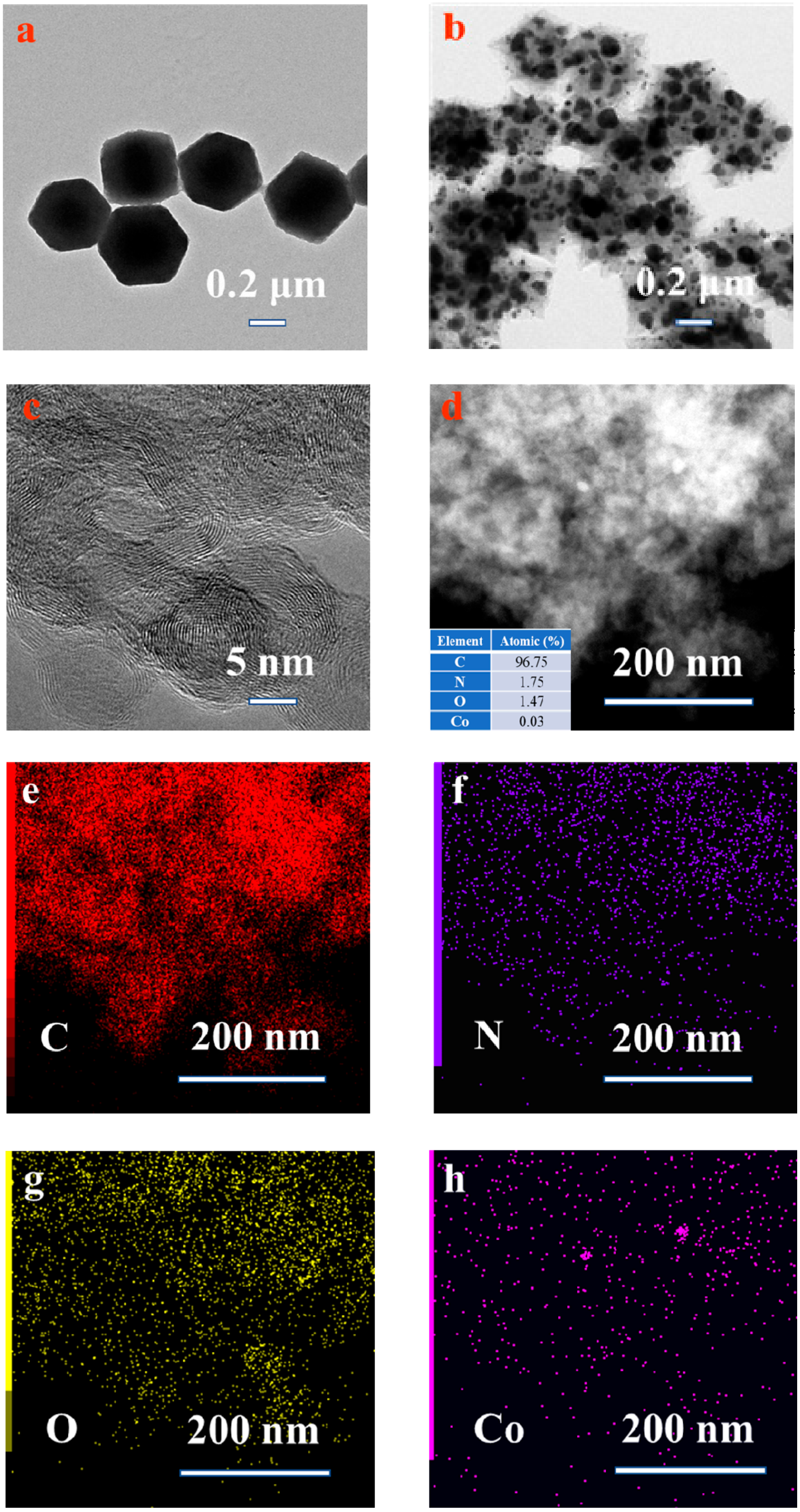

Figure S2 TEM images of (a) ZIF-67 and (b) NPC-900; (c) HRTEM image of O-NPC-120. (d) STEM-mapping images and the EDS data of O-NPC-120. (e) - (h) EDS of O-NPC-120.

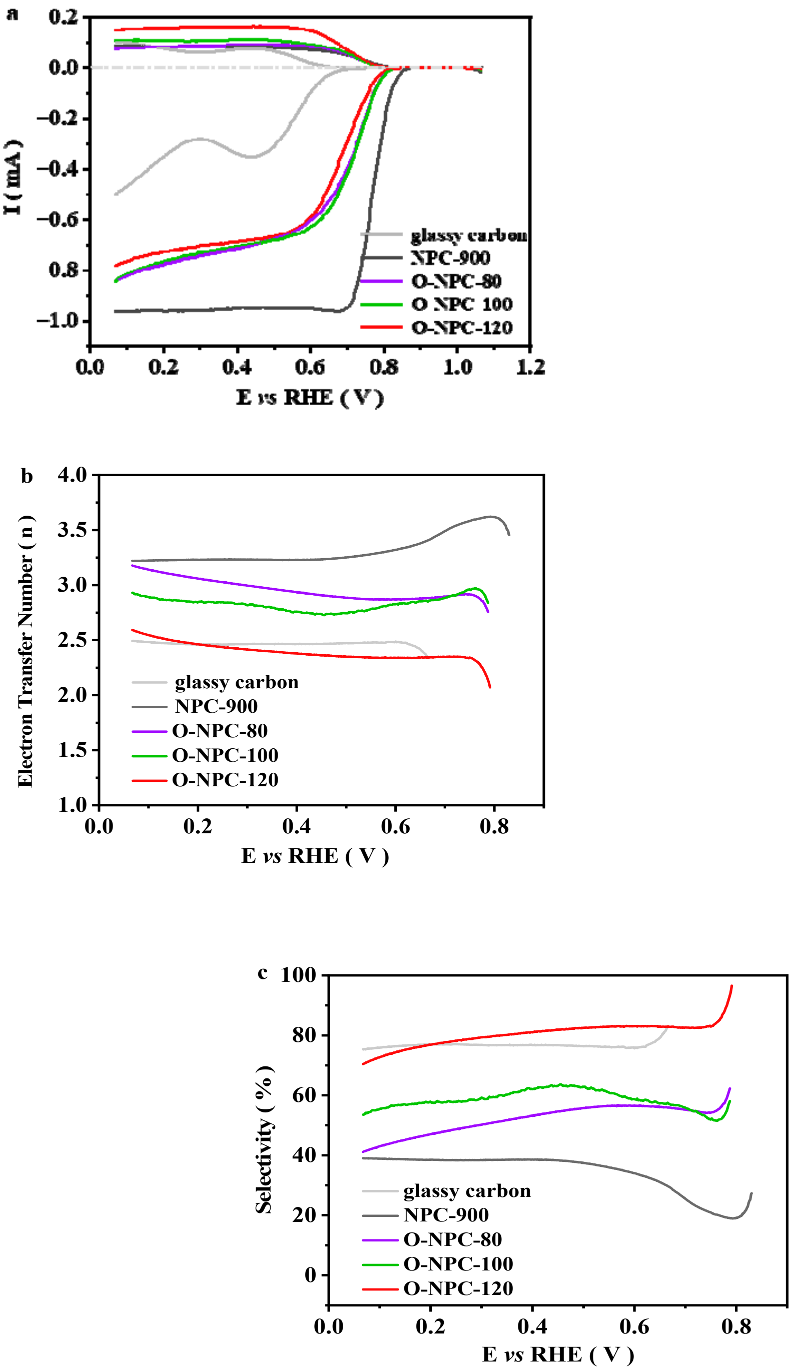

Figure S3 Comparison of $2e^-$ ORR performance between glassy carbon electrode (GCE) and catalyst loading GCE. (a) RRDE polarization curve. (b) electron transfer number. (c) H_2O_2 selectivity.

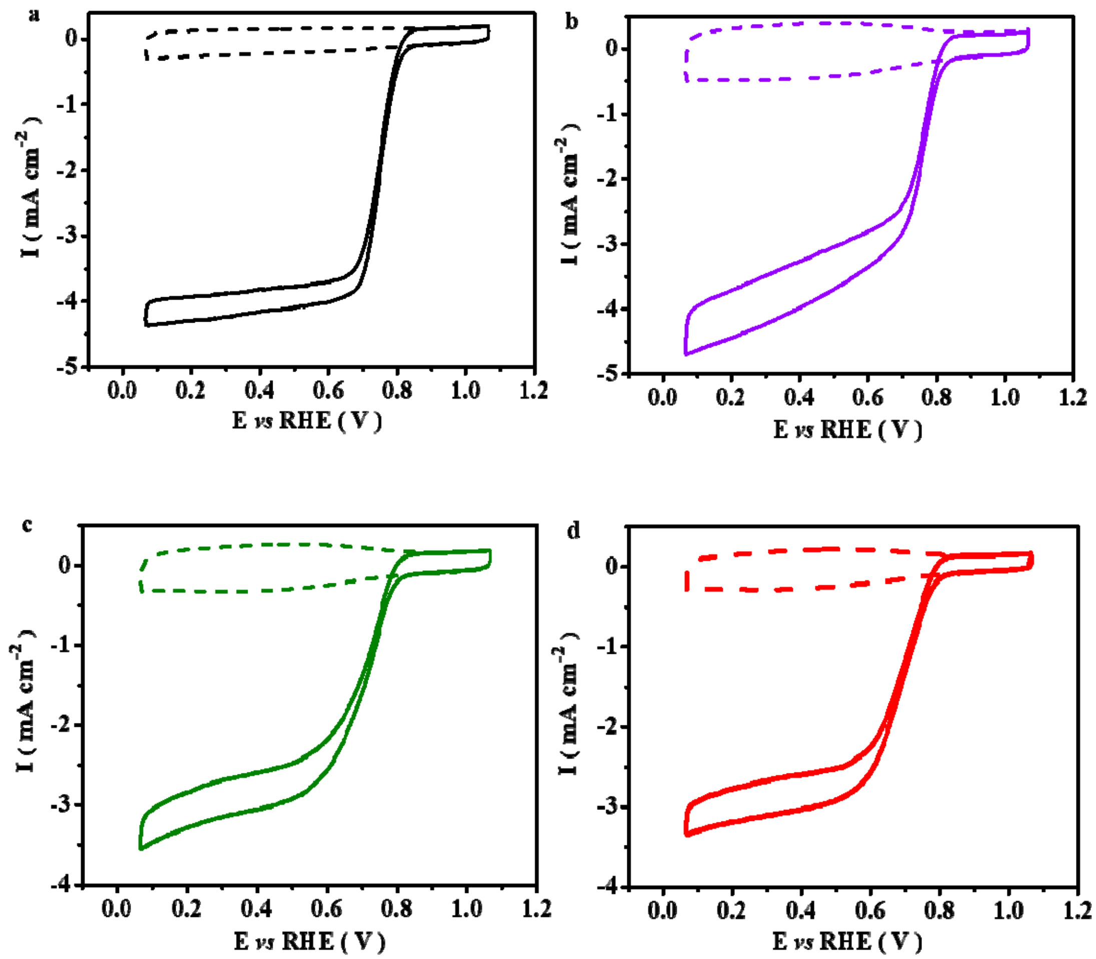

Figure S4 CV curves of the catalysts at 1600 rpm and 50 mV s^{-1} in N_2- and O_2-saturated electrolyte. (a) NPC-900. (b) O-NPC-80. (c) O-NPC-100. (d) O-NPC-120. All dashed lines: N_2; all solid lines: O_2.

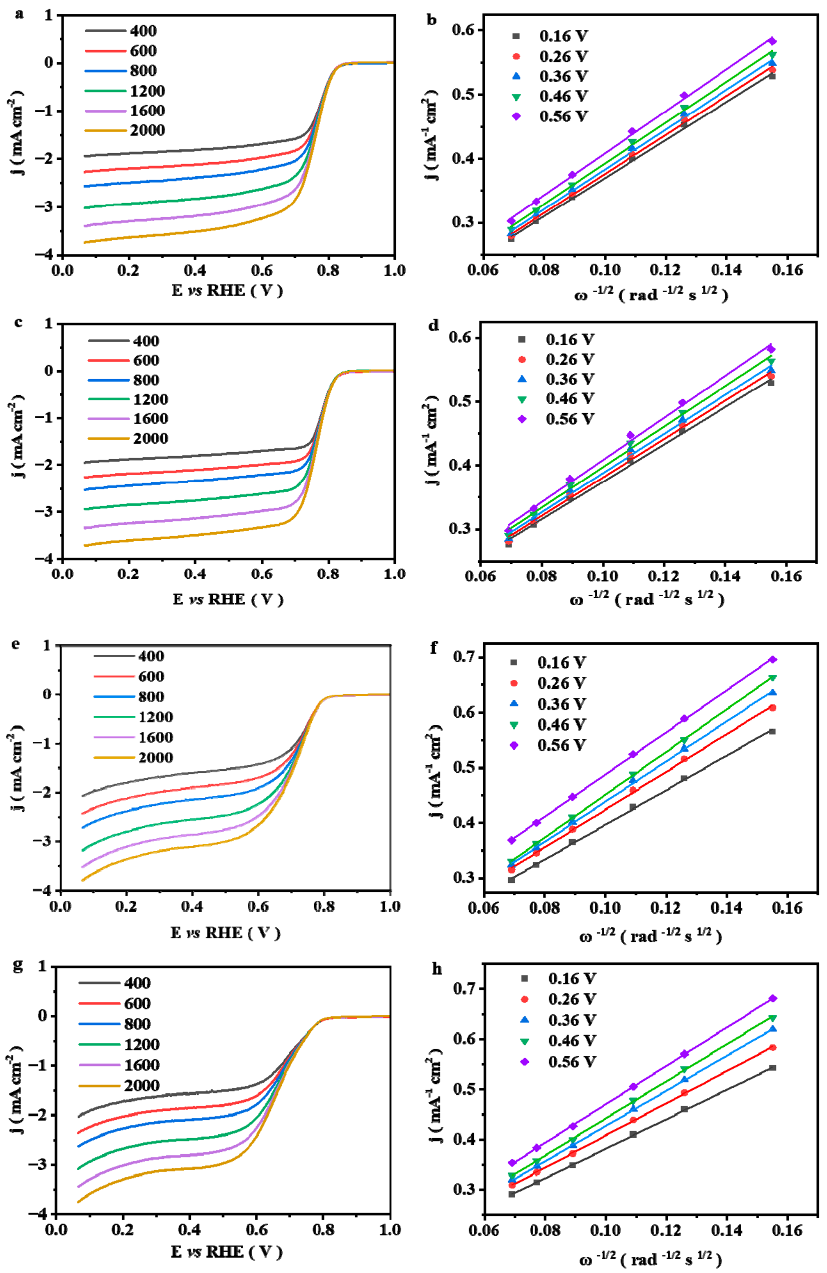

Figure S5 LSV curves of (a) NPC-900, (c) O-NPC-80, (e) O-NPC-100, (g) O-NPC-120 measured at different rotation speeds in O_2 saturated 0.1 M KOH. (b) NPC-900, (d) O-NPC-80, (f) O-NPC-100, (h) O-NPC-120 are Koutecky-Levich plots based on corresponding LSV curves.

The electron transfer number was further calculated through the K-L equation. The n value is 3.22, 3.05, 2.85 and 2.51 for NPC-900, O-NPC-80, O-NPC-100 and O-NPC-120, respectively. These n values are consistent with the measured results of RRDE.

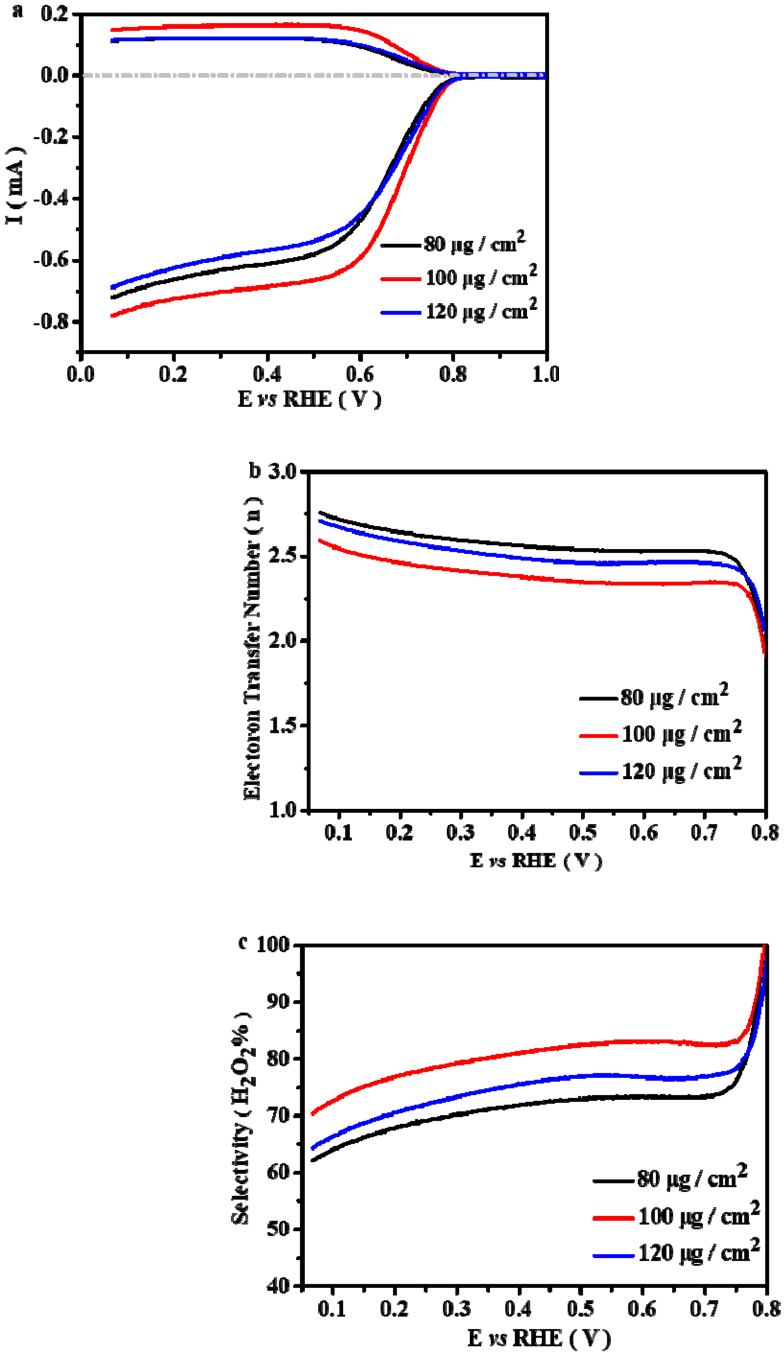

Figure S6 Effect of O-NPC-120 loading on catalytic performance of 2e$^-$ ORR. (a) RRDE polarization curve. (b) electron transfer number. (c) H_2O_2 selectivity.

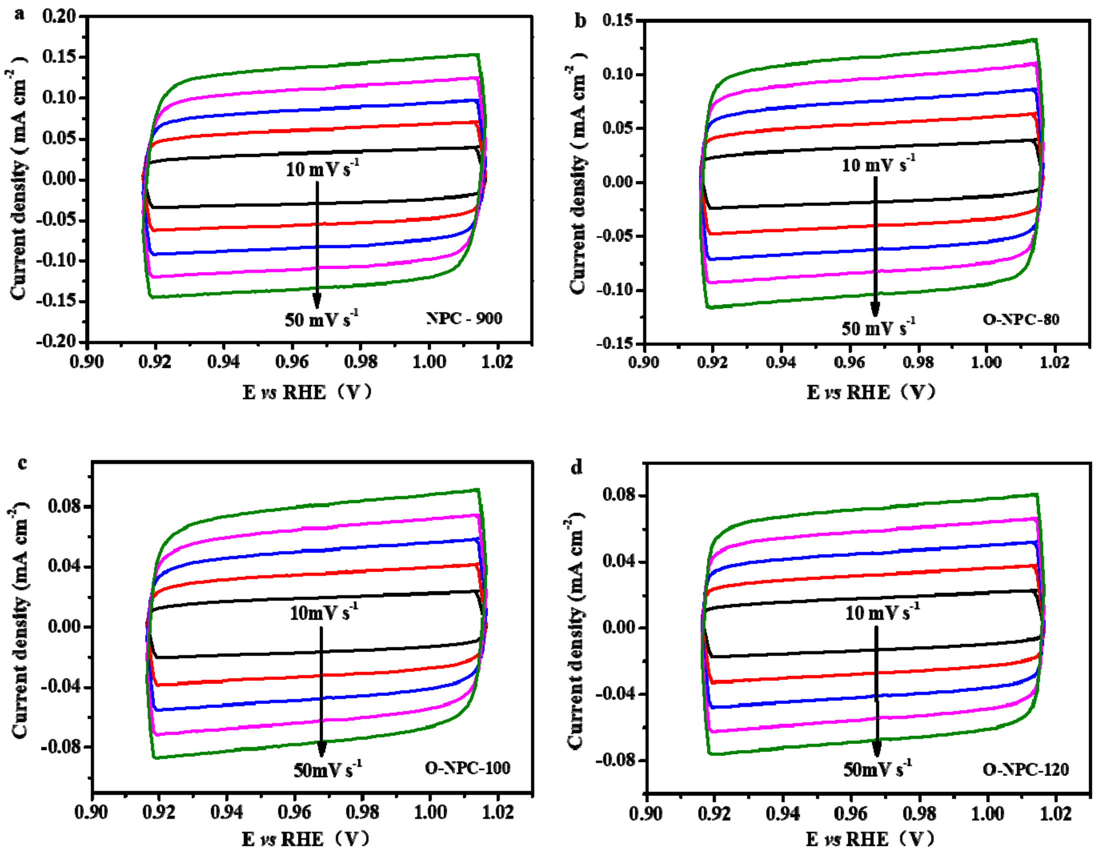

Figure S7 Cyclic voltammogram in the non-faradic potential region at varying scan rates for the samples of (a) NPC-900. (b) O-NPC-80. (c) O-NPC-100. (d) O-NPC-120.

C_{dl} is obtained from Figure S3 after calculation with the following equation:

$$C_{dl} = (Ja - Jc) / 2$$

Where, *Ja* is the capacitance current density at 0.963V (vs. RHE) during voltage forward scanning, *Jc* is the capacitance current density at 0.963V (vs. RHE) during negative voltage scanning.

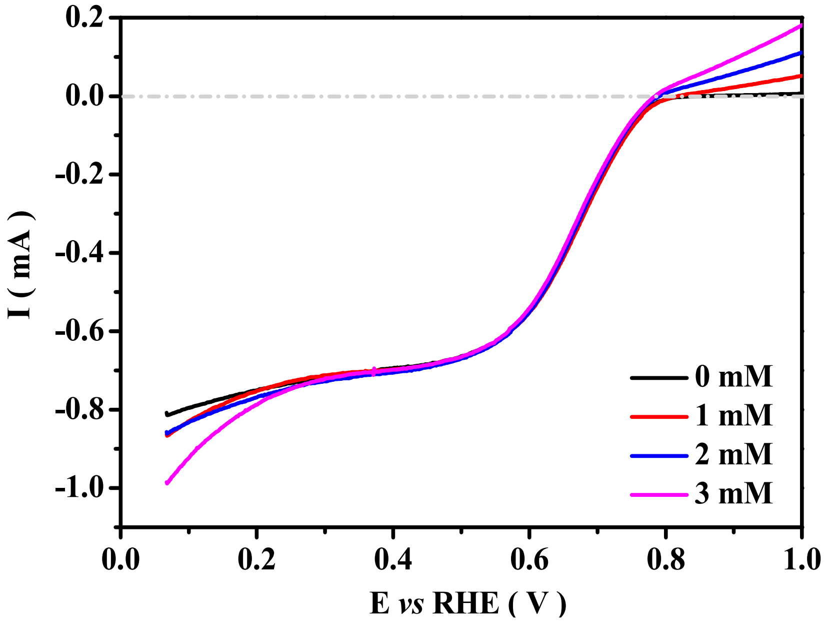

Figure S8 LSV curves of O-NPC-120 in 0.1M KOH solution with H_2O_2 concentrations of 0 mM, 1 mM, 2 mM, and 3 mM.

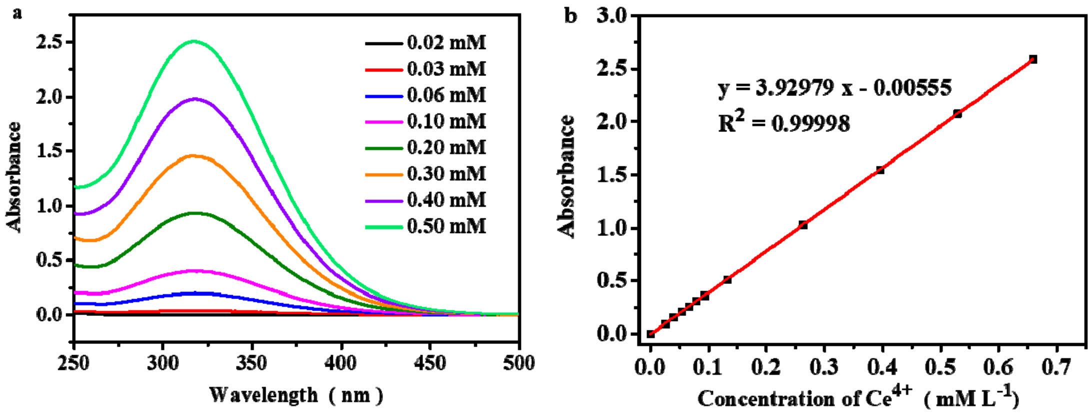

Figure S9 (a) UV-Vis spectra of Ce^{4+} solutions with different concentrations. (b) is the standard curve corresponding to (a).

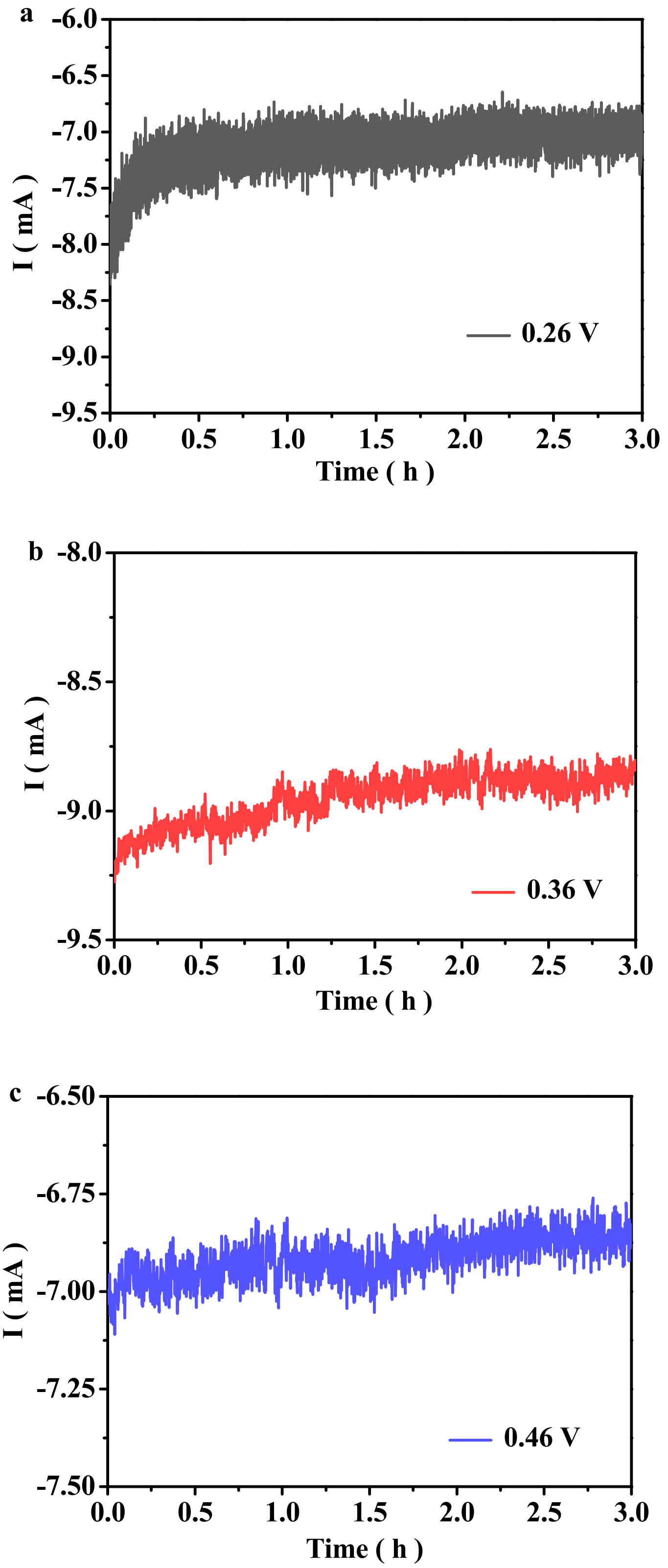

Figure S10 *i-t* curves of O-NPC-120 in 0.1 M KOH electrolyte under different voltages. (a) 0.26 V. (b) 0.36 V. (c) 0.46 V. Loading capacity of catalyst: 100 μg / cm^2.

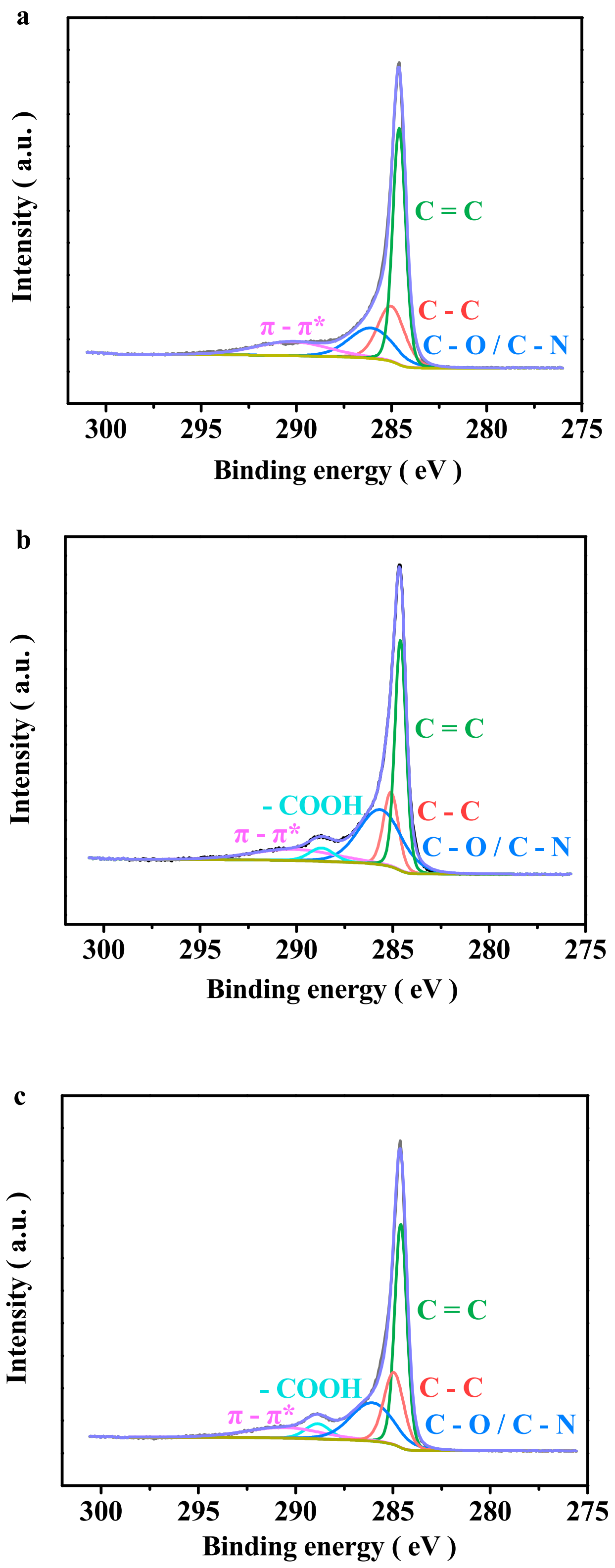

Figure S11 The XPS-characterized C 1s spectra of (a) NPC-900, (b) O-NPC-80 and (c) O-NPC-100, respectively.

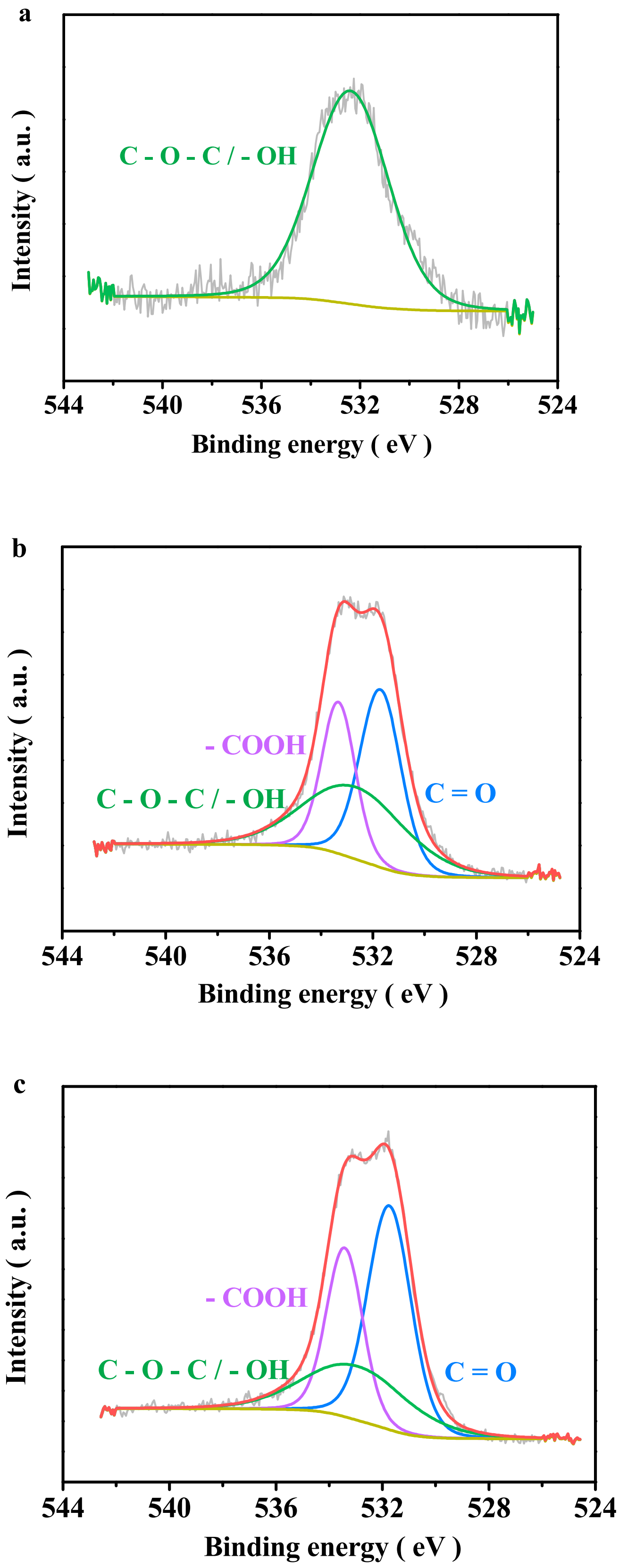

Figure S12 O 1s spectra characterized by XPS (a) NPC-900, (b) O-NPC-80 and (c) O-NPC-100, respectively.

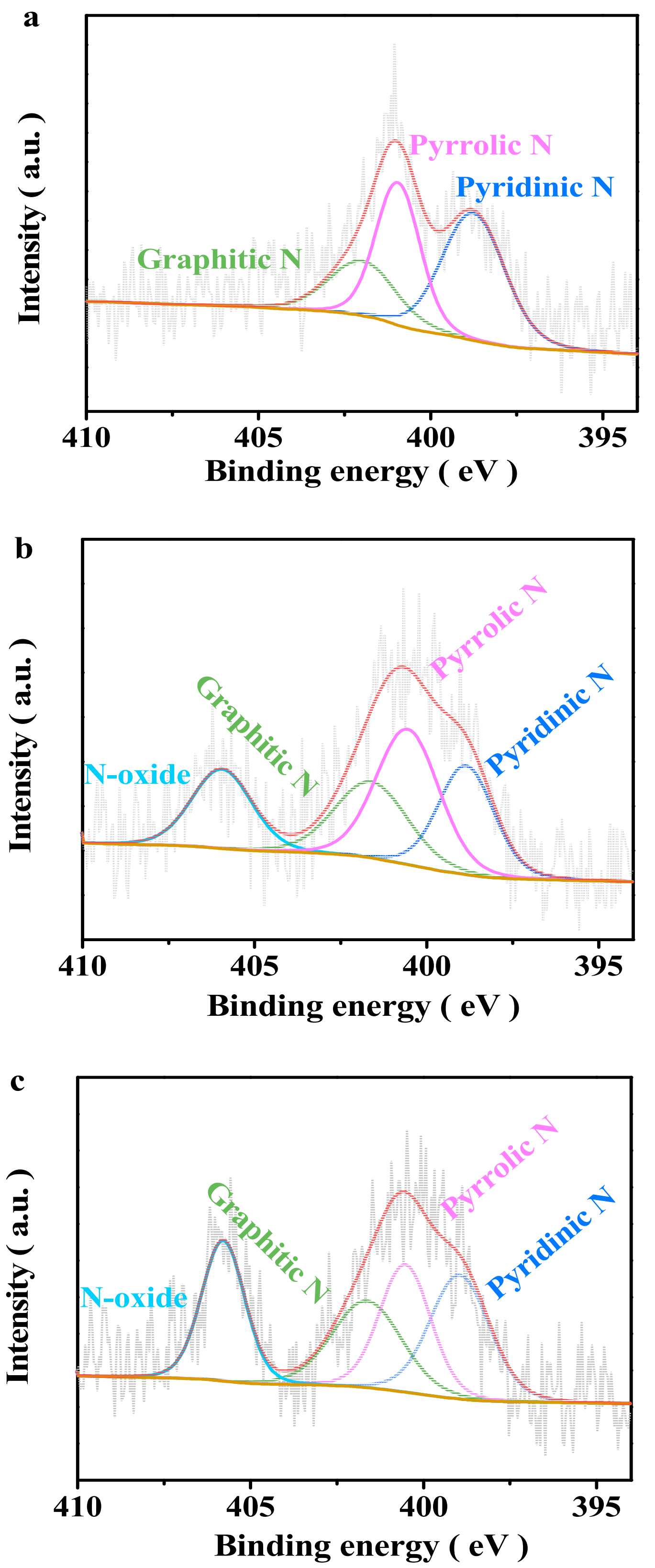

Figure S13 N 1s spectra characterized by XPS (a) NPC-900, (b) O-NPC-80 and (c) O-NPC-100, respectively.

Table S1. C, N, O contents of catalysts detected by XPS

Sample	Peak BE (eV)	Atomic (%)	element
NPC-900	284.60	95.59	C 1s
	401.28	1.25	N 1s
	531.60	3.16	O 1s
O-NPC-80	284.60	87.39	C 1s
	402.43	1.04	N 1s
	531.60	11.57	O 1s
O-NPC-100	284.60	86.25	C 1s
	402.87	1.09	N 1s
	531.60	12.66	O 1s
O-NPC-120	284.60	84.15	C 1s
	402.88	1.02	N 1s
	531.60	14.53	O1s

Table S2. Different C types and contents in catalysts determined from XPS analysis results

Sample	Peak BE (eV)	Atomic (%)	Carbon states
NPC-900	284.60	44.07	sp^2 C: C = C
	285.01	21.82	sp^3 C: C - C
	286.06	19.40	C-O / C-N
	290.59	14.71	$\pi \rightarrow \pi^*$
O-NPC-80	284.60	35.13	sp^2 C: C = C
	285.10	15.27	sp^3 C: C - C
	286.05	32.37	C-O / C-N
	288.89	4.50	- COOH
	290.62	12.73	$\pi \rightarrow \pi^*$
O-NPC-100	284.60	40.72	sp^2 C: C = C
	285.05	21.15	sp^3 C: C - C
	286.06	21.26	C-O / C-N
	288.86	5.41	- COOH
	290.59	11.46	$\pi \rightarrow \pi^*$
O-NPC-120	284.60	32.01	sp^2 C: C = C
	285.06	25.29	sp^3 C: C - C
	286.02	25.10	C-O / C-N
	288.89	5.95	- COOH
	290.63	11.65	$\pi \rightarrow \pi^*$

Table S3. Different O types and contents in catalysts determined from XPS analysis results

Sample	Peak BE (eV)	Atomic (%)	Oxygen states
NPC-900	532.39	100	C - O – C / - OH
O-NPC-80	531.71	37.08	C = O
	532.66	37.18	C - O – C / - OH
	533.37	25.74	- COOH
O-NPC-100	531.74	45.22	C = O
	532.75	26.21	C - O – C / - OH
	533.40	28.58	- COOH
O-NPC-120	531.76	43.50	C = O
	532.77	25.43	C - O – C / - OH
	533.38	31.07	- COOH

Table S4. Different N types and contents in catalysts determined from XPS analysis results

Sample	Peak BE (eV)	Atomic (%)	element
NPC-900	398.76	44.45	Pyridinic N
	400.95	35.22	Pyrrolic N
	401.98	20.33	Graphitic N
O-NPC-80	398.85	24.44	Pyridinic N
	400.56	34.22	Pyrrolic N
	401.61	22.30	Graphitic N
	405.95	19.04	N-oxide
O-NPC-100	398.96	28.44	Pyridinic N
	400.53	25.10	Pyrrolic N
	401.63	23.16	Graphitic N
	405.79	23.30	N-oxide
O-NPC-120	398.94	25.96	Pyridinic N
	400.56	25.63	Pyrrolic N
	401.63	26.05	Graphitic N
	405.71	22.36	N-oxide

Table S5 $2e^-$ ORR performance of some carbon-based catalysts

Catalyst	electrolyte	Onset potential [V_{RHE}]	Selectivity [%]	Tafel [mV dec^{-1}]	Productivity	Ref
O-NPC-120	0.1 M KOH	0.79	83.1	45.16	2909.79 mmol $g_{catalyst}^{-1}$ h^{-1}	This work
N-MCs	0.1 M KOH	—	85	——	——	[1]
Meso-C	0.1 M KOH	~ 0.7	~ 100	——	——	[2]
OXO-G/ $NH_3 \cdot H_2O$	0.1 M KOH	~ 0.8	＞80	——	224.8 mmol $g_{catalyst}^{-1}$ h^{-1} at 0.2 V_{RHE} (H type cell)	[3]
N-FLG-8	0.1 M KOH	0.80	＞95	——	9.66 mol $g_{catalyst}^{-1}$ h^{-1} (flow cell, 1.8V)	[4]
NOC-6M	0.1 M KOH		95.2	59		[5]
CB-Plasma	0.1 M KOH		100	——		[6]
GOMC	0.1 M KOH	0.78	＞90	48	24 mM (H type cell: 16 h)	[23]
CB + CTAB	0.1 M KOH	0.80	＞95	~ 60	——	[62]
NT-3DFG	0.1 M KOH	0.79 ± 0.01	＞94	54.8 ± 1.8	——	[63]
CNT-F-0.6	0.05 M Na_2SO_4 pH = 7		82 ~ 95	258	7.40 mmol g^{-1} h^{-1} (GDE: 300 mL undivided cell)	[64]
HCNFs	0.1 M KOH	＞0.80	＞89	75.6	45864 mM g^{-1} h^{-1} [flow cell (two-compartment cell)]	[65]
B-C	0.1 M KOH	0.773	＞85	78（1M KOH）	14720 mmol g^{-1} h^{-1}(solid-electrolyte cell)	[66]

N-CMK3IL	0.5 M H_2SO_4		＞95	——	159.9 mmol $g_{catalyst}^{-1}$ h^{-1} at 0.1 V_{RHE} (H type cell)	[71]
	0.1 M K_2SO_4		＞83	——	547.07 mmol $g_{catalyst}^{-1}$ h^{-1} at 0.2 V_{RHE} (H type cell)	
	0.1 M KOH		＞85	——	561.7 mmol $g_{catalyst}^{-1}$ h^{-1} at 0.1 V_{RHE} (H type cell)	
NPCNS	0.1 M KOH	—	＞85	67.8	223.4 mmol $g_{catalyst}^{-1}$ h^{-1} at 0.3 V_{RHE} (H type cell)	[72]
N-doped C	0.1 M KOH	0.88 V	93	——		[73]
CMK3	0.1 M KOH	~ 0.80	90	——	——	[74]
O-CNT	0.1 M KOH		＞85	47	111.71 mmol g^{-1} h^{-1}	[75]
BNC	0.1 M KOH	0.80	＞80	——	——	[76]

References

[1] X. Sheng, N. Daems, B. Geboes, M. Kurttepeli, S. Bals, T. Breugelmans, A. Hubin, I. F. Vankelecom, P. P. Pescarmona, N-doped ordered mesoporous carbons prepared by a two-step nanocasting strategy as highly active and selective electrocatalysts for the reduction of O_2 to H_2O_2, Appl. Catal. B 176 (2015) 212 - 224.

[2] S. Chen, Z. Chen, S. Siahrostami, T. R. Kim, D. Nordlund, D. Sokaras, S. Nowak, J. W. To, D. Higgins, R. Sinclair, T. Jaramillo, J. Nørskov, Z. Bao, Defective Carbon-Based Materials for the Electrochemical Synthesis of Hydrogen Peroxide, ACS Sustainable Chem. Eng. 6 (2018) 311 - 317.

[3] L. Han, Y. Y. Sun, S. Li, C. Cheng, C. E. Halbig, P. Feicht, J. L. Hübner, P. Strasser, S. Eigler, In-Plane Carbon Lattice-Defect Regulating Electrochemical Oxygen Reduction to Hydrogen Peroxide Production over Nitrogen-Doped Graphene, ACS Catal. 9 (2019) 1283 - 1288.

[4] L. Li, C. Tang, Y. Zheng, B. Q. Xia, X. L. Zhou, H. L. Xu, S. Z. Qiao, Tailoring Selectivity of Electrochemical Hydrogen Peroxide Generation by Tunable Pyrrolic-Nitrogen-Carbon, Adv. Energy Mater. 10 (2020) 852 - 863.

[5] C. Y. Zhang, G. Z. Liu, B. Ning, S. R. Qian, D. N. Zheng, L. Wang, Highly efficient electrochemical generation of H_2O_2 on N / O co-modified defective carbon, Int. J. Hydrog. Energy 46 (2021) 14277 - 14287.

[6] Z. Wang, Q. K. Li, C. H. Zhang, Z. H. Cheng, W. Y. Chen, E. A. McHugh, R. A. Carter, B. I. Yakobson, J. M. Tour, Hydrogen Peroxide Generation with 100% Faradaic Efficiency on Metal-Free Carbon Black, ACS Catal. 11 (2021) 2454 - 2459.
